# Commentary: Plant-based diet and risk of all-cause mortality: a systematic review and meta-analysis

**DOI:** 10.3389/fnut.2025.1529857

**Published:** 2025-01-31

**Authors:** William B. Grant

**Affiliations:** Sunlight, Nutrition, and Health Research Center, San Francisco, CA, United States

**Keywords:** all-cause mortality, cancer mortality, cardiovascular mortality, cohort study, meta-analysis, follow-up period, healthy plant-based diet, unhealthy plant-based diet

## Introduction

The recent article on plant-based diets and risk of all-cause mortality by Tan et al. (1) provides the basis to examine the effect of underestimating the real associations of mortality rates (regression dilution) due to changes in the values of the dietary variables with time after their determination at baseline. Changes in variables were demonstrated in 1999 using data from the Framingham Study in the US (2). Figure 1 in that article shows paired mean values for blood pressure and cholesterol for quintiles defined at baseline and remeasured every two years. The range for the first and fifth quintile for systolic blood pressure changed from 51 mmHg at baseline to 35 mmHg at 6 years, 27 mmHg at 16 years, and 18 mmHg at 26 years. Thus, health outcomes at longer follow-up periods will be based on values that gradually depart from baseline values.

Regression dilution has been demonstrated for various health outcomes with respect to serum 25-hydroxyviamin D [25(OH)D] concentrations. The most recent review shows the effect for stroke and major cardiovascular (CVD) events with respect to baseline serum 25(OH)D concentrations for follow-up periods of one–to–ten and one–to-eight years, respectively (3). This review also references earlier reviews for all-cause mortality rate, cancer incidence, incidence of Alzheimer's disease and dementia. The analyses generally show that the value of regression fit to the shortest follow-up period is about twice the size of the standard meta-analysis that does not take follow-up period into account.

## Analysis

For the analysis mean follow-up periods were obtained from the data in Table 1 in Tan et al. (1). Values for the effect of plant-based diets were obtained from Figures 2–4 in Tan et al. (1). Data were analyzed using SigmaStat 4.0 (Grafiti, Palo Alto, CA, USA). The RR values for diet in the four-year follow-up study were based on a comparison of the 90th percentile with the 10th percentile of PDI (4). The hazard ratio values were converted to those for quintiles to align with the 7.8-year follow-up period study, which was based on the extreme quintiles.

**Table 1:** Regression fits to the relative risks of healthy plant-based diets and unhealthy plant-based diets and cancer, CVD, and all-cause mortality rates based on data in Tan et al. (1).

**Outcome**	**Type of diet**	**N**	**Follow-up periods (years)**	**Regression equation for RR; RR (95% CI) for four years**	***r, adjr^2^, p***	**Tan et al. (1) RR (95% CI)**
Cancer mortality	hPDI	7	4–28	0.78 + 0.009 × years; 0.79 (0.71–0.92)	0.92, 0.86, 0.002	0.91 (0.83–0.99)
		3	4–10	0.67 + 0.026 × years; 0.77 (0.68–0.86)	0.96, 0.85, 0.18	
	uPDI	7	4–28	1.26 – 0.009 × years; 1.22 (1.09–1.36)	0.63, 0.27, 0.13	1.10 (0.97–1.26)
		5	4–22	1.31 – 0.016 × years; 1.25 (1.10–1.41)	0.91, 0.78, 0.03	
		3	4–10	1.40 – 0.030 × years; 1.28 (1.17–1.39)	0.81, 0.31, 0.40	
CVD mortality	hPDI	6	4–25	0.87+0.002 × years; 0.88 (0.77–1.02)	0.28, 00, 0.59	0.85 (0.77–0.94)
		3	4–10	0.61+ 0.042 × years; 0.78 (0.71–0.86)	0.64, 00, 0.56	
	uPDI	6	4–25	1.46 – 0.018 × years; 1.39 (1.15–1.63)	0.81, 0.58, 0.048	1.19 (1.07–1.32)
		5	4–22	1.43 – 0.015 × years; 1.37 (1.15–1.63)	0.71, 0.34, 0.18	
		3	4–10	1.64 – 0.046 × years; 1.46 (1.30–1.64)	0.76, 0.11, 0.46	
All–cause mortality	hPDI	10	4–28	0.73 + 0.008 × years; 0.77 (0.71–0.84)	0.69, 0.41, 0.03	0.85 (0.80–0.90)
		4	4–10	0.50 + 0.045 × years; 0.68 (0.64–0.73)	0.94, 0.83, 0.06	
	uPDI	10	4–28	1.36 – 0.012 × years; 1.31 (1.21–1.43)	0.69, 0.41, 0.03	1.18 (1.09–1.27)
		4	4–10	1.36 – 0.009 × years; 1.32 (1.24–1.41)	0.22, 0.00, 0.78	

CVD, cardiovascular disease; hPDI, healthy plant-based diet; N, number of studies; RR, relative risk; uPDI, unhealthy plant-based diet.

Table 1 gives the equations for relative risk (RR), the statistical correlation results and *p*-values, and the RR values calculated by Tan et al. In the analyses, follow-up periods were used from four–to–ten to 28 years. Since follow-up periods longer than 10 years result in significant reductions in correlations between variables and health outcomes, different maximum follow-up periods were used in the analyses reported in Table 1.

The values of the regression fit for the shortest follow-up period, 4 years, is considered to be the most accurate correlation between the diet and the mortality rates. As can be seen, the difference from 1.00 for RRs for 4-year values for follow-up periods up to 25 years from 0.8 to 2.3 times as large as for Tan's RR; for 4-year values for follow-up periods <10 years from about 1.5 to 2.8 times as large as for Tan's RR. Also, that the regression fits to the data show that using the cutoff for the longest follow-up period at >20 years generally decreases the calculated RR for four years for hPDI. These findings are attributed to omitting studies where the “regression dilution” effect is greater. However, the *p*-value is mostly non-significant when fewer studies being used in the analysis.

The regression fits to the data for CVD are the least robust. This may be due to the fact that mortality from CVD often occurs after a number of years with CVD, resulting in various approaches to treatment.

## Discussion

A recent Harvard analysis of diet and incidence of various diseases was published (5). The data for the analysis were obtained from 205,852 healthcare professionals from three US cohorts followed for up to 32 years. Participants completed food frequency questionnaires every 4 years. As shown in Table 2 of that article, the fully-adjusted associations between the 90th and 10th percentiles for the hPDI were 0.84 (95% CI, 0.82–0.87) for major chronic diseases; 0.85 (95% CI, 0.81–0.90) for major CVD; 0.78 (95% CI, 0.75–0.81) for type 2 diabetes mellitus; and 0.95 (95% CI, 0.91–0.99) for cancer. It is well known that vitamin D has a much larger effect on cancer mortality rates than on cancer incidence rates (6). Perhaps the same thing applies to diet as well.
